# Segment selection for fusion and artificial disc replacement in the hybrid surgical treatment of noncontiguous cervical spondylosis: a finite element analysis

**DOI:** 10.3389/fbioe.2024.1345319

**Published:** 2024-04-03

**Authors:** Xiangyao Sun, Jiang Huang, Qingming Zhang, Li Cao, Yuqi Liu, Zelong Song, Wei Tang, Siyuan Sun, Juyong Wang

**Affiliations:** ^1^ Department of Orthopaedics, Xuanwu Hospital, Capital Medical University, Beijing, China; ^2^ National Clinical Research Center for Geriatric Diseases, Beijing, China; ^3^ Beijing Glitzern Technology Co., Ltd., Beijing, China; ^4^ Department of Emergency, Xuanwu Hospital, Capital Medical University, Beijing, China; ^5^ Nankai University School of Medicine, Nankai University, Tianjin, China; ^6^ Department of Orthopaedics, The PLA General Hospital, Beijing, China; ^7^ Department of Interdisciplinary, Life Science, Purdue University, West Lafayette, IN, United States

**Keywords:** cervical degenerative disease, interior cervical discectomy, adjacent segment disease, cervical disc arthroplasty, hybrid surgery, finite element analysis

## Abstract

**Introduction:** The treatment of skip-level cervical degenerative disease (CDD) with no degenerative changes observed in the intervening segment (IS) is complicated. This research aims to provide a reference basis for selecting treatment approaches for noncontiguous CDD.

**Methods:** To establish accurate finite element models (FEMs), this study included computed tomography (CT) data from 21 patients with CDD (10 males and 11 females) for modeling. The study primarily discusses four cross-segment surgical approaches: upper (C3/4) anterior cervical discectomy and fusion (ACDF) and lower (C5/6) cervical disc arthroplasty (CDA), FA model; upper CDA (C3/4) and lower ACDF (C5/6), AF model; upper ACDF (C3/4) and lower ACDF (C5/6), FF model; upper CDA (C3/4) and lower CDA (C5/6), AA model. An initial axial load of 73.6 N was applied at the motion center using the follower load technique. A moment of 1.0 Nm was applied at the center of the C2 vertebra to simulate the overall motion of the model. The statistical analysis was conducted using STATA version 14.0. Statistical significance was defined as a *p* value less than 0.05.

**Results:** The AA group had significantly greater ROM in flexion and axial rotation in other segments compared to the FA group (*p* < 0.05). The FA group consistently exhibited higher average intervertebral disc pressure in C2/3 during all motions compared to the AF group (*p* < 0.001); however, the FA group displayed lower average intervertebral disc pressure in C6/7 during all motions (*p* < 0.05). The AA group had lower facet joint contact stresses during extension in all segments compared to the AF group (*p* < 0.05). The FA group exhibited significantly higher facet joint contact stresses during extension in C2/3 (*p* < 0.001) and C6/7 (*p* < 0.001) compared to the AF group.

**Discussion:** The use of skip-level CDA is recommended for the treatment of non-contiguous CDD. The FA construct shows superior biomechanical performance compared to the AF construct.

## 1 Introduction

Anterior cervical discectomy (ACDF) is a commonly performed surgical procedure for the treatment of single or multilevel cervical degenerative disease (CDD). It has been proven to have high fusion rates and favorable clinical outcomes (Wu et al., 2017a). However, ACDF carries the risk of segmental instability and adjacent segment disease (ASD) (Xiong et al., 2018). This is attributed to the biomechanical changes induced by ACDF, including reduced range of motion (ROM) at the surgical segment, altered endplate and intradiscal stress, and increased load on the facet joints, which may contribute to adjacent segment degeneration (Wang et al., 2018; Xiong et al., 2018).

On the other hand, cervical disc arthroplasty (CDA) aims to preserve the physiological motion between adjacent vertebrae, thus avoiding abnormal stress transfer to the adjacent segments (Lee et al., 2012). CDA mimics the natural coupling of cervical spine motion by preserving the intervertebral instant center of rotation (ICR) (Kaiser et al., 2002; Laratta et al., 2018). Both ACDF and CDA achieve effective neural decompression and restoration of intervertebral disc height, contributing to successful treatment of CDD (Zhao et al., 2015). The management of long-segment CDD is more complex compared to short-segment CDD. This is particularly true when treating skip-level CDD, where there are no degenerative changes observed in the intervening segment (IS), posing significant challenges in selecting the appropriate surgical approach (Sun et al., 2020). Previous research has highlighted the importance of preserving the IS to enhance the effectiveness of skip-level CDD treatment (Qizhi et al., 2016; Shi et al., 2016; Wu et al., 2017b). Therefore, the surgical approach should aim to maintain the normal biomechanical properties of the IS. Long-segment ACDF is not recommended for noncontiguous skip-level CDD as it can disrupt the natural intervertebral disc structure of the IS (Wu et al., 2017a). Moreover, long-segment ACDF is associated with a higher risk of internal fixation-related complications, including implant failure and the development of ASD (Yang et al., 2016). Although noncontiguous ACDF preserves the integrity of the IS, it leads to increased postoperative motion and stress on the IS, thereby elevating the risk of IS degeneration (Sun et al., 2020). Previous studies have demonstrated the superiority of cervical disc arthroplasty (CDA) over ACDF in the treatment of two-level CDD, as it maintains cervical mobility and stability of adjacent segments (Zhao et al., 2015; Wu et al., 2017b). Furthermore, compared to single-level CDA, multi-level CDA has shown more favorable clinical outcomes and functional recovery without a higher incidence of complications (Goffin et al., 2003; Sun et al., 2020). However, multi-level CDA is associated with various challenges such as heterotopic ossification, prolonged surgical time, increased intraoperative bleeding, suboptimal cervical alignment, vertebral body fracture, and prosthetic displacement (Coric et al., 2011). These factors limit the application of noncontiguous CDA in the management of non-contiguous CDD (Liu et al., 2015).

The Hybrid surgery, which combines CDA and ACDF techniques, has been proposed as a potential solution. The concept behind Hybrid surgery is to preserve cervical mobility while reducing abnormal loads on adjacent segments, thus potentially preventing the occurrence of ASD (Wu et al., 2019a). However, the actual therapeutic efficacy of Hybrid surgery is still a matter of debate, and the precise surgical indications for its use have not been clearly defined, leading to ongoing discussions regarding its clinical application (Wu et al., 2019b). Previous studies have suggested that the use of Hybrid surgery in long-segment cervical procedures can effectively prevent postoperative complications associated with multi-level ACDF, while preserving cervical mobility (Barbagallo et al., 2009). Therefore, Hybrid surgery may be an ideal approach for treating noncontiguous CDD. However, further discussions are needed regarding the specific surgical strategy formulation and selection of surgical indications.

Finite element analysis (FEA) is a commonly employed biomechanical research method that can enhance our understanding of cervical biomechanical characteristics. By utilizing finite element modeling based on cervical anatomy parameters, we can visually represent the biomechanical effects of different surgical treatment methods (Kallemeyn et al., 2010). However, previous studies have often relied on a single standard model to compare different treatment methods for CDD, without considering individual variations among patients (Manickam and Roy, 2022). Therefore, the primary objective of this study is to incorporate multiple patients with noncontiguous CDD into finite element analysis, enabling modeling and analysis.

The study aims to simulate noncontiguous skip-level ACDF, CDA, and various types of Hybrid surgeries. Statistical methods will be employed to analyze the data and compare the biomechanical characteristics of different treatment methods. This research endeavors to provide a reference basis for selecting appropriate treatment approaches for noncontiguous CDD.

## 2 Materials and methods

### 2.1 Finite element modelling

To create precise finite element models (FEMs), this study utilized computed tomography (CT) data from 21 patients diagnosed with cervical degenerative disease (CDD), comprising 10 males and 11 females. The CT images were obtained using a SOMATOM Definition AS + scanner (Siemens, Germany) with a thickness of 0.75 mm and an interval of 0.69 mm. These radiological images served as the basis for constructing realistic clinical models of the cervical spine (C2∼C7) through the application of FEA techniques (Sun et al., 2023). The geometric models of the C2∼C7 cervical spine were reconstructed using Mimics 17.0 software (Materialize Inc., Leuven, Belgium) from the radiological images. Subsequently, these geometric models were exported as STL files and further refined into physical structures using Geomagic Studio 12.0 software (3D Systems Corporation, Rock Hill, SC, United States). This meticulous process allowed for the creation of detailed and reliable finite element models, which served as the foundation for the subsequent simulation and analysis of various surgical treatment methods for noncontiguous CDD.

In previous research, the focus on noncontiguous cervical spine diseases has mainly centered around the middle segment C4/5 (Wu et al., 2019a; Sun et al., 2020; Sun et al., 2023). However, for the purpose of this study, the attention was directed towards the C3/4 and C5/6 segments for surgical procedures. Four cross-segment surgical approaches were primarily explored: the upper (C3/4) ACDF and lower (C5/6) cervical disc arthroplasty (CDA) in the FA model; upper CDA (C3/4) and lower ACDF (C5/6) in the AF model; upper ACDF (C3/4) and lower ACDF (C5/6) in the FF model; and upper CDA (C3/4) and lower CDA (C5/6) in the AA model. These surgical strategies were simulated using the aforementioned software.

**FIGURE 1:**
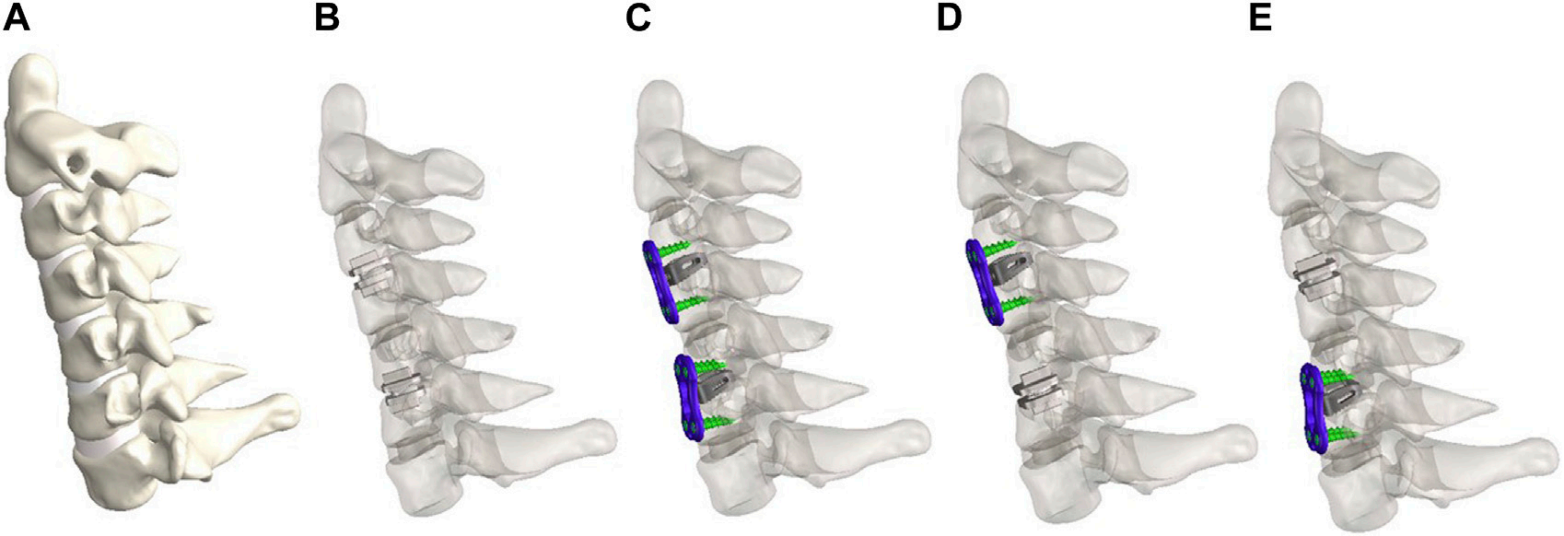
FEMs of intact group **(A)**, CDA-CDA (AA) group **(B)**, ACDF-ACDF (FF) group **(C)**. ACDF-CDA (FA) group **(D)** and CDA-ACDF (AF) group **(E)**.

In the ACDF approach, the relevant intervertebral disc, anterior longitudinal ligament (ALL), and posterior longitudinal ligament (PLL) were removed, and NuVasive^®^ Helix ACP and CoRoent^®^ Contour implants were inserted at the intervertebral level. The analyzed implants in this study included NuVasive^®^ Helix ACP, CoRoent^®^ Contour, and Synthes^®^ Prodisc-C (Table 1). The FEMs of these devices were constructed using Solidworks 2016 and integrated with the cervical spine models. On the other hand, in the CDA approach, the relevant intervertebral connections were removed, and Prodisc-C was implanted at the corresponding level (Figure 1). High-quality meshes of these models were generated using Hypermesh 12.0. Subsequently, material properties and experimental conditions were defined, followed by setting up the finite element analysis in ABAQUS 6.13. These steps ensured the accurate representation and analysis of the various surgical approaches for noncontiguous CDD in this study.

**TABLE 1:** Dimensions and material parameters of devices.

Devices	NuVasive^®^ Helix ACP	CoRoent^®^ Contour	Synthes^®^ Prodisc-C		
Dimensions	Plate: 16 mm long, 24 mm wide and 2.4 mm thick	17 mm long, 14 mm wide, 6 mm high, 7° lordotic	16 mm long, 15 mm wide, 6 mm high		
	Screws: diameter of 4.5mm, 14 mm long				
Materials	Ti6Al4V	PEEK	Cobalt chromium (end plates)		Polyethylene (core)
Elastic modulus (MPa)	114.000	3,400	210,000		800
Poisson Ratio	0.35	0.4	0.3		0.3

PEEK, polyether-ether-ketone.

### 2.2 Material properties

The material properties and element types used in the finite element models are presented in Table 2 (Mo et al., 2017; Sun et al., 2020; Tohamy et al., 2022). The cancellous bone was represented as tetrahedral elements (C3D4) with a mesh size of 3 mm and was established based on the solid volume of the vertebrae. The cortical bone, which covered the cancellous bone, had a uniform thickness of 0.4 mm and was represented by triangular shell elements (S3) with nodes coinciding with the exterior surface of the cancellous bone. The intervertebral disc was composed of the nucleus pulposus and annulus fibrosus. The fibrous annulus contained a group of crossed fibers that only experienced tension, constituting approximately 19% of the fibrous annulus. The nucleus pulposus, making up about 40% of the intervertebral disc, was located near its center (Mo et al., 2015). The annulus ground substance and nucleus pulposus were meshed using hexahedral elements (C3D8R), while the annulus fibers were represented by tension-only truss elements (T3D2). For the facet joints, the articular surfaces were covered by cartilage, and the surface contact was considered nonlinear. The cartilage thickness and gap were both set at 0.5 mm (Wu et al., 2019b). Additionally, the ALL, PLL, ligamentum flavum (LF), interspinous ligament (IL), supraspinous ligament (SL), and capsular ligament (CL) were inserted into the model as six groups of ligaments and were defined as T3D2 elements (Tang et al., 2022). These elements and properties were chosen to accurately represent the biomechanical characteristics of the cervical spine and its associated structures in the finite element analysis.

**TABLE 2:** Material parameters of the cervical spine.

Material	Elastic modulus (MPa)	Poisson ratio	Cross-sectional area (mm^2^)
Vertebra			
Cortical bone	12,000	0.29	-
Cancellous bone	100	0.29	-
Endplate	1,200	0.29	-
Cartilage	10.4	0.4	-
Posterior structure	3,500	0.25	-
Intervertebral disc			
Annulus fiber	450	0.45	-
Annulus ground substance	3.4	0.4	-
Nucleus pulposus	1	0.49	-
Ligaments			
ALL	30	0.4	12
PLL	20	0.4	45
LF	10	0.4	14
CL	10	0.4	5
IL	10	0.4	13.1

ALL, anterior longitudinal ligament; PLL, posterior longitudinal ligament; LF, ligament flavum; IL, interspinous ligament; CL, capsular ligament.

### 2.3 Boundary and loading conditions

To ensure proper alignment, constraints were imposed on the interfaces between the natural structures of the cervical spine. The interaction between the facet joints was modeled as sliding friction, allowing for realistic contact behavior. The lower endplate of the C7 vertebra was fully restricted in all directions to ensure stability during the analysis (Manickam and Roy, 2022). The connections between the screws and the vertebral bodies, as well as between the screws and the implanted device, were treated as fixed constraints to simulate their rigid attachment. The interface between the cancellous bone graft material and the CoRoent^®^ Contour implant was defined as having no friction to enable smooth interaction. Furthermore, the endplates and intermediate layer of the ProDisc-C implant were considered fully bonded, providing a strong connection (Lin et al., 2009; Sun et al., 2020). In the finite element analysis, an initial axial load of 73.6 N was applied at the motion center using the follower load technique to replicate the effects of muscle forces and the weight of the head. Additionally, a moment of 1.0 Nm was applied at the center of the C2 vertebra to simulate the overall motion of the C2∼C7 finite element model, including flexion, extension, lateral bending, and axial rotation. Throughout the analysis, the range of motion (ROM) for each intervertebral segment was calculated and compared against existing data to validate the accuracy and reliability of the finite element model (Lee et al., 2016; Liu et al., 2016; Wu et al., 2019a; Sun et al., 2020; Sun et al., 2023). These procedures were essential to accurately simulate the biomechanical behavior of the cervical spine and to obtain reliable results for the different surgical treatment methods used in the study.

### 2.4 Statistical analysis

The statistical analysis was performed using STATA version 14.0 (Stata Corp LP, College Station, Texas, United States). Continuous variables were presented as mean ± standard deviations (SD). The normality of continuous data was assessed using the Kolmogorov-Smirnov test. For normally distributed data, either one-way analysis of variance (ANOVA) or Student’s t-test was utilized for analysis. In the case of skewed distributed data, the Kruskal–Wallis test was applied. A significance level of *p* < 0.05 was considered statistically significant in all analyses.

## 3 Results

### 3.1 Validation of finite element models

**FIGURE 2:**
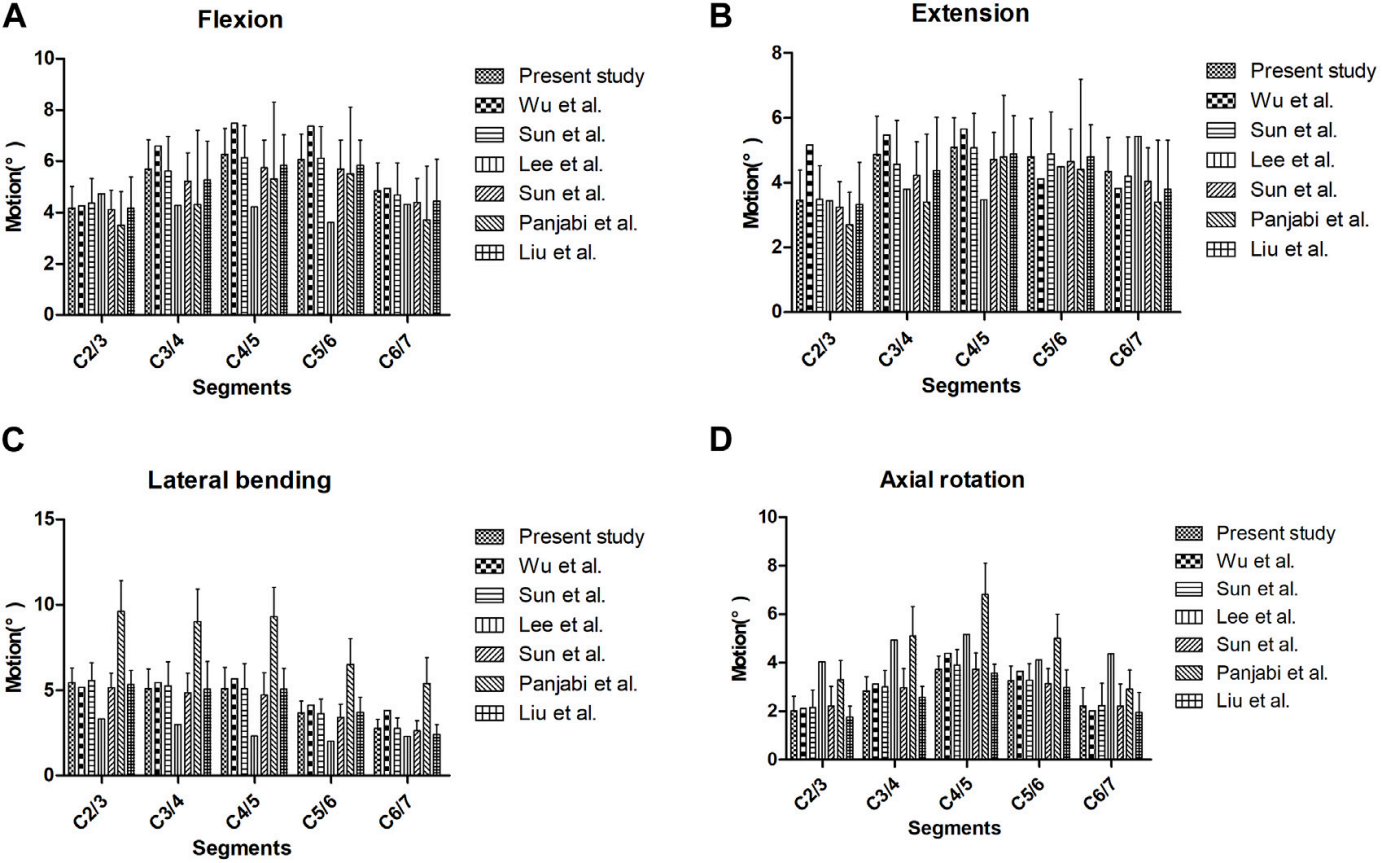
The ROM for each segment of the cervical spine finite element model under different motion conditions in different articles: **(A)** flexion motion, **(B)** extension motion, **(C)** lateral bending, and **(D)** axial rotation.

In this study, the range of motion (ROM) for each segment of the complete C2∼C7 finite element model was compared to data from previous studies. The findings showed a strong concordance between the average ROM of each segment in this study and the results reported in existing literature, providing validation for the reliability and accuracy of the C2∼C7 finite element model utilized in this investigation (Figure 2) (Panjabi et al., 2001; Lee et al., 2016; Liu et al., 2016; Wu et al., 2019b; Sun et al., 2020; Sun et al., 2023).

### 3.2 Comparison of ROMs

Table 3 present the comparative results of ROM in each segment among different groups during various motions. When comparing the AA group to the FA group, there was no significant difference in ROM during various motions in C2/3 (*p* > 0.05). However, the AA group exhibited significantly greater ROM in flexion and axial rotation in other segments compared to the FA group (*p* < 0.05). When comparing the AA group to the AF group, the AA group showed significantly higher ROM during various motions in the IS (C4/5), lower surgical segment (C5/6), and lower segment (C6/7) compared to the AF group (*p* < 0.05). In the comparison between the FA group and the AF group, the FA group demonstrated significantly higher ROM in the lower segment (C6/7) during extension compared to the AF group (*p* < 0.001), while no significant difference was observed in ROM for other motions (*p* > 0.05). Moreover, ROM in the IS was significantly higher in the FA group compared to the AF group during extension and lateral bending (*p* = 0.001). In C2/3, the FA group had significantly lower ROM during lateral bending compared to the AF group (*p* < 0.001), while for other motions, the ROM in the FA group was significantly higher than in the AF group (*p* < 0.05).

**TABLE 3:** Comparison of ROMs at different intervertebral levels.

Motion	Segments	ROM				*p* Values					
		FF	AA	FA	AF	FF vs. AA	FF vs. FA	FF vs. AF	AA vs. FA	AA vs. AF	FA vs. AF
Flexion	C2/3	7.98 ± 1.05	4.45 ± 0.86	4.04 ± 0.78	3.56 ± 0.61	<0.001	<0.001	<0.001	0.116	<0.001	0.037
	C3/4	1.00 ± 0.11	6.35 ± 0.82	0.45 ± 0.08	6.04 ± 1.17	<0.001	<0.001	<0.001	<0.001	0.329	<0.001
	C4/5	12.97 ± 1.30	7.37 ± 0.83	5.62 ± 0.99	5.50 ± 1.03	<0.001	<0.001	<0.001	<0.001	<0.001	0.709
	C5/6	1.22 ± 0.23	7.21 ± 0.83	1.69 ± 0.90	1.26 ± 0.23	<0.001	0.029	0.523	<0.001	<0.001	0.048
	C6/7	8.62 ± 0.95	5.29 ± 0.67	4.51 ± 0.78	0.23 ± 0.72	<0.001	<0.001	<0.001	<0.001	<0.001	0.200
Extension	C2/3	5.15 ± 0.97	3.05 ± 0.61	2.97 ± 0.67	2.38 ± 0.58	<0.001	<0.001	<0.001	0.701	0.001	0.005
	C3/4	0.87 ± 0.20	4.94 ± 0.59	0.53 ± 0.12	5.09 ± 1.06	<0.001	<0.001	<0.001	<0.001	0.575	<0.001
	C4/5	10.59 ± 1.34	5.36 ± 0.80	5.40 ± 1.28	4.14 ± 0.95	<0.001	<0.001	<0.001	0.900	<0.001	0.001
	C5/6	0.83 ± 0.16	4.73 ± 0.82	4.42 ± 1.08	0.58 ± 0.14	<0.001	<0.001	<0.001	0.307	<0.001	<0.001
	C6/7	5.72 ± 1.00	4.04 ± 0.67	4.81 ± 1.12	3.40 ± 0.80	<0.001	0.010	<0.001	0.012	0.009	<0.001
Lateral bending	C2/3	5.64 ± 0.78	2.95 ± 0.77	3.28 ± 0.65	4.19 ± 0.74	<0.001	<0.001	<0.001	0.149	<0.001	<0.001
	C3/4	0.82 ± 0.10	5.36 ± 0.85	0.65 ± 0.11	3.88 ± 0.81	<0.001	<0.001	<0.001	<0.001	<0.001	<0.001
	C4/5	11.22 ± 1.00	7.17 ± 0.92	6.04 ± 1.19	4.88 ± 0.91	<0.001	<0.001	<0.001	0.002	<0.001	0.001
	C5/6	0.65 ± 0.06	5.56 ± 0.90	5.12 ± 0.91	0.69 ± 0.12	<0.001	<0.001	0.193	0.136	<0.001	<0.001
	C6/7	4.20 ± 0.67	3.28 ± 0.72	2.61 ± 0.53	2.72 ± 0.55	<0.001	<0.001	<0.001	0.002	0.009	0.534
Axial rotation	C2/3	4.25 ± 0.32	1.97 ± 0.33	2.28 ± 0.45	1.88 ± 0.40	<0.001	<0.001	<0.001	0.019	0.409	0.005
	C3/4	0.98 ± 0.36	3.26 ± 0.40	0.51 ± 0.10	3.01 ± 0.56	<0.001	<0.001	<0.001	<0.001	0.108	<0.001
	C4/5	5.34 ± 0.44	3.98 ± 0.35	3.43 ± 0.69	3.57 ± 0.68	<0.001	<0.001	<0.001	0.003	0.021	0.537
	C5/6	1.00 ± 0.31	3.70 ± 0.35	3.19 ± 0.60	0.54 ± 0.11	<0.001	<0.001	<0.001	0.002	<0.001	<0.001
	C6/7	3.31 ± 0.47	1.88 ± 0.37	1.64 ± 0.36	1.53 ± 0.30	<0.001	<0.001	<0.001	0.045	0.002	0.290

ROM, range of motion; AA, double arthroplasty; AF, upper arthroplasty and lower fusion; FA, upper fusion and lower arthroplasty; FF, double fusion.

### 3.3 Comparison of average intervertebral disc pressures

Table 4 present the results of between-group comparisons of average intervertebral disc pressure in each segment during various motions. When compared to the AF group, the AA group showed lower average intervertebral disc pressure in other segments during various motions (*p* < 0.05), except for extension in C2/3, where no significant between-group difference was observed (*p* = 0.550). Additionally, the FA group consistently exhibited higher average intervertebral disc pressure in C2/3 during all motions compared to the AF group (*p* < 0.001). However, the FA group displayed lower average intervertebral disc pressure in C6/7 during all motions (*p* < 0.05).

**TABLE 4:** Comparison of average pressures in intervertebral discs.

Motion	Segments	Average pressures				*p* Values					
		FF	AA	FA	AF	FF vs. AA	FF vs. FA	FF vs. AF	AA vs. FA	AA vs. AF	FA vs. AF
Flexion	C2/3	0.34 ± 0.04	0.23 ± 0.03	0.35 ± 0.06	0.26 ± 0.06	<0.001	0.571	<0.001	<0.001	0.024	<0.001
	C4/5	0.41 ± 0.06	0.27 ± 0.03	0.44 ± 0.09	0.42 ± 0.08	<0.001	0.130	0.846	<0.001	<0.001	0.220
	C6/7	0.46 ± 0.04	0.27 ± 0.03	0.30 ± 0.30	0.43 ± 0.09	<0.001	<0.001	0.303	0.034	<0.001	<0.001
Extension	C2/3	0.36 ± 0.06	0.24 ± 0.03	0.35 ± 0.08	0.23 ± 0.05	<0.001	0.576	<0.001	<0.001	0.550	<0.001
	C4/5	0.40 ± 0.05	0.26 ± 0.04	0.47 ± 0.09	0.43 ± 0.10	<0.001	0.008	0.364	<0.001	<0.001	0.176
	C6/7	0.47 ± 0.03	0.28 ± 0.03	0.29 ± 0.07	0.41 ± 0.10	<0.001	<0.001	0.009	0.473	<0.001	<0.001
Lateral bending	C2/3	0.55 ± 0.06	0.35 ± 0.02	0.59 ± 0.11	0.40 ± 0.10	<0.001	0.217	<0.001	<0.001	0.043	<0.001
	C4/5	0.63 ± 0.07	0.41 ± 0.04	0.66 ± 0.14	0.63 ± 0.15	<0.001	0.473	0.972	<0.001	<0.001	0.561
	C6/7	0.70 ± 0.05	0.41 ± 0.03	0.48 ± 0.11	0.56 ± 0.10	<0.001	<0.001	<0.001	0.019	<0.001	0.017
Axial rotation	C2/3	0.62 ± 0.04	0.38 ± 0.03	0.62 ± 0.13	0.43 ± 0.10	<0.001	0.842	<0.001	<0.001	0.029	<0.001
	C4/5	0.68 ± 0.07	0.43 ± 0.05	0.73 ± 0.13	0.69 ± 0.15	<0.001	0.129	0.792	<0.001	<0.001	0.360
	C6/7	0.72 ± 0.05	0.44 ± 0.04	0.47 ± 0.10	0.59 ± 0.11	<0.001	<0.001	<0.001	0.180	<0.001	0.002

AA, double arthroplasty; AF, upper arthroplasty and lower fusion; FA, upper fusion and lower arthroplasty; FF, double fusion.

### 3.4 Comparison of contact forces in cervical facet joints

Table 5 present the results of between-group comparisons of the contact stresses in facet joints during extension. The AA group showed significantly lower facet joint contact stresses during extension in C2/3 (*p* < 0.001) and C4/5 (*p* < 0.001) compared to the FA group. However, the AA group had significantly higher facet joint contact stresses during extension in C6/7 compared to the FA group (*p* < 0.001). Additionally, the AA group had lower facet joint contact stresses during extension in all segments compared to the AF group (*p* < 0.05). On the other hand, the FA group exhibited significantly higher facet joint contact stresses during extension in C2/3 (*p* < 0.001) and C6/7 (*p* < 0.001) compared to the AF group.

**TABLE 5:** Comparison of average contact forces in facet joints in extension.

Segments	Average contact forces (N)				*p* Values					
	FF	AA	FA	AF	FF vs. AA	FF vs. FA	FF vs. AF	AA vs. FA	AA vs. AF	FA vs. AF
C2/3	106.13 ± 9.95	62.62 ± 6.25	99.88 ± 10.57	84.58 ± 8.92	<0.001	0.061	<0.001	<0.001	<0.001	<0.001
C4/5	118.16 ± 7.44	87.44 ± 9.50	106.23 ± 10.89	106.72 ± 11.03	<0.001	<0.001	<0.001	<0.001	0.005	0.889
C6/7	99.52 ± 7.99	83.45 ± 5.97	75.76 ± 7.85	106.37 ± 11.36	<0.001	0.032	0.023	<0.001	<0.001	<0.001

AA, double arthroplasty; AF, upper arthroplasty and lower fusion; FA, upper fusion and lower arthroplasty; FF, double fusion.

## 4 Discussion

The management of multilevel CDD remains a subject of controversy. ACDF offers advantages such as direct lesion removal, excision of degenerated intervertebral discs, and correction of cervical curvature (Lee et al., 2011). However, when using long-segment ACDF to address non-contiguous CDD, it can disrupt the physiological function and structural stability of the intervening normal segments. As a result, long-segment ACDF is not considered an ideal approach for treating non-contiguous CDD. Skip-level ACDF, on the other hand, may lead to the development of ASD, with reported incidence rates ranging from 6.25% to 20% in short-term follow-ups. Over the long term, the intervening segments can undergo gradual degeneration due to excessive motion and increased stress (Wu et al., 2017a). Similarly, in our study, skip-level ACDF significantly increased the range of motion and facet joint contact stress in adjacent segments compared to other surgical techniques. Therefore, skip-level ACDF is not recommended as an optimal treatment approach for non-contiguous CDD.

Previous research has demonstrated that skip-level CDA is a safe and effective approach for treating noncontiguous CDD. However, the indications for multi-level CDA are more stringent, resulting in higher surgical complexity, reduced postoperative cervical stability, and an increased risk of implant-related complications, leading to higher overall costs (Wu et al., 2017b). Previous studies have shown that skip-level CDA does not significantly increase intervertebral disc pressure or facet joint pressure in the intervening segment compared to adjacent segments (Wu et al., 2017a). Similarly, in our study, skip-level CDA did not result in significantly higher adjacent segment disc stress compared to the intact cervical spine, suggesting that skip-level CDA can play a role in preventing ASD. The design of artificial discs aims to mimic the biomechanical characteristics of natural discs, and the rotational center of the artificial disc should align as closely as possible with the rotational center of the cervical motion segment. Therefore, the rotational center of artificial discs is commonly located behind the inferior endplate of the lower vertebral body (Lin et al., 2009). While the artificial nucleus of an artificial disc can maintain disc height, it still requires good fixation with the endplate screws to ensure stress transmission along the axis of the cervical spine (Wu et al., 2019a). If the elastic potential energy cannot dissipate through the artificial nucleus within the artificial disc, a significant amount of stress may be exerted on the bony contact surfaces of the endplates. This is particularly evident during flexion motion, where a large amount of stress can be transmitted to the adjacent segments, thereby increasing the risk of ASD (Lin et al., 2009; Sun et al., 2023). In our study, skip-level CDA resulted in increased facet joint contact stress in the intermediate and lower segments during extension. This phenomenon may be related to the placement of the artificial disc’s rotational center. To achieve the optimal position of the artificial disc’s rotational center, its implantation position is relatively posterior, which increases the stress burden on the posterior column of the adjacent segment. As a result, there is an increased risk of posterior column structural degeneration in the intermediate and lower segments. Although skip-level CDA effectively reduces intervertebral pressure in adjacent segments, it comes at the cost of decreased overall cervical stiffness and, consequently, decreased overall cervical stability (Lee et al., 2016).

Hybrid surgery is considered a relatively safe and effective approach in the treatment of multilevel cervical spondylosis, as it combines the advantages of both ACDF and CDA techniques. The Hybrid construct achieves a balance between stability and mobility, allowing for preserved cervical mobility without a significant reduction in cervical stiffness (Coric et al., 2011). As a result, its application is becoming increasingly common in clinical practice (Panjabi et al., 2001). The selection of segments for ACDF and CDA in Hybrid surgery plays a crucial role in determining the overall structural biomechanical performance. In a Hybrid construct, the choice of the lower ACDF segment can significantly impact the stress distribution on the lower segments (Lee et al., 2011). However, in this study, we found no significant difference in intervertebral disc stress and facet joint contact stress in the IS between the FA group (upper ACDF and lower CDA) and the AF group (upper CDA and lower ACDF), suggesting that both Hybrid surgical approaches offer similar protective effects on the IS. This indicates that both Hybrid surgeries can effectively preserve the biomechanical integrity of the IS.

Hybrid surgery is considered a relatively safe and effective approach in the treatment of multilevel cervical spondylosis, as it combines the advantages of both ACDF and CDA techniques. The Hybrid construct achieves a balance between stability and mobility, allowing for preserved cervical mobility without a significant reduction in cervical stiffness (Wu et al., 2019b). As a result, its application is becoming increasingly common in clinical practice (Barrey et al., 2012). The selection of segments for ACDF and CDA in Hybrid surgery plays a crucial role in determining the overall structural biomechanical performance. In a Hybrid construct, the choice of the lower ACDF segment can significantly impact the stress distribution on the lower segments (Li et al., 2018). However, in this study, we found no significant difference in intervertebral disc stress and facet joint contact stress in the IS between the FA group (upper ACDF and lower CDA) and the AF group (upper CDA and lower ACDF), suggesting that both Hybrid surgical approaches offer similar protective effects on the IS. This indicates that both Hybrid surgeries can effectively preserve the biomechanical integrity of the IS.

Hybrid surgery may lead to an overall increase in cervical facet joint contact stress, potentially influencing the long-term effectiveness of the surgery (Li et al., 2018). In this study, we observed that the facet joint contact stress in the upper and lower adjacent segments was lower in the FA group (upper ACDF and lower CDA) compared to the AF group (upper CDA and lower ACDF), suggesting that the FA group provides better protection for the posterior column structures. This could be attributed to the upper ACDF in the FA group, which corrects the upper cervical curvature, resulting in a more ideal direction of force transmission and relatively reduced stress on the posterior column structures. After Hybrid surgery, the cervical spine requires greater torque to achieve full range of motion compared to an intact spine. However, this increased torque can cause fatigue of the paraspinal muscles during cervical motion, posing a risk to the long-term mobility of the cervical spine (Li et al., 2018). The study findings suggest that ACDF may be more suitable for segments with smaller range of motion in Hybrid surgery, while CDA may be more suitable for segments with larger range of motion to minimize the impact on adjacent segments (Sun et al., 2023). By carefully considering the biomechanical characteristics of each surgical approach, surgeons can make more informed decisions in choosing the appropriate Hybrid surgery strategy for individual patients, thereby optimizing the long-term outcomes of the surgery.

Several clinical studies have indicated that both Hybrid surgery and skip-level CDA have a preventive effect on ASD and offer protection for the IS (Wu et al., 2017a). However, the findings from this study revealed that skip-level CDA exhibited an overall better protective effect on adjacent segments and IS compared to Hybrid surgery. The reason for this discrepancy lies in the clinical practice, where doctors often choose Hybrid surgery for patients with well-preserved IS, as ACDF can lead to stress concentration on adjacent segments. On the other hand, CDA can better mimic the functional characteristics of a normal intervertebral disc, making it more suitable for patients with mild degeneration in adjacent segments. The preoperative condition of adjacent segments can influence the assessment of ASD occurrence postoperatively. By utilizing biomechanical methods to systematically analyze different treatment approaches, this study effectively eliminated the interference of confounding factors that are commonly encountered in clinical studies, thereby improving the accuracy and reliability of the results. Biomechanical analysis offers valuable insights into the specific effects of each surgical approach on cervical biomechanics, enabling a more comprehensive understanding of their advantages and limitations. By integrating biomechanical findings with clinical evidence, surgeons can make more informed decisions when selecting the appropriate surgical treatment for patients with non-contiguous cervical degenerative disease, thus optimizing the long-term outcomes and enhancing patient care.

This study has several limitations that should be acknowledged. Firstly, to enhance the credibility of the finite element model, CT data from patients with CDD were utilized. However, accurately modeling degenerated structures, such as degenerated intervertebral discs, and assigning appropriate material properties to these structures presented challenges. Garay et al. (Garay et al., 2022) stated that the bone mineral density (BMD) of different cervical vertebrae segments and parts was different due to various physiological loads. In order to reduce the impact of BMD at different cervical vertebrae levels on the study results, the surgical levels were the same for all models in this study. In addition, this study focused on analyzing the biomechanical properties of the three-joint complex at each movement level of the cervical spine, and did not discuss the stress and strain of the bone structure. Therefore, the structure of the cervical spine model is simplified into a columnar structure composed of bone cortex and bone cancellum in the front and a uniform arcuated posterior unit structure in the rear, which is helpful to save computing resources and improve the feasibility of the study, so as to make the study more targeted. Consequently, the comparison of different surgeries was conducted within an ideal analytical environment. The simplified model is difficult to reflect the complex coupling motion between intervertebral disc and facet joint. In addition, the pulling action of muscles is also difficult to simulate in finite element models. In this study, the overall properties of the cervical spine models were adjusted by adjusting the elastic modulus of the intervertebral discs and the elastic modulus of the ligaments, so that its biomechanical properties were close to the physiological state of the complete cervical spine. Therefore, after simplifying the cervical spine models, this study made them consistent with the data provided by previous published literatures as far as possible in terms of validity verification, so as to improve the credibility of the models. Although the cervical spine model’s validity was verified, and statistical analysis was applied to the data, enhancing the representativeness and credibility of the research results to some extent, it partially addressed the aforementioned limitations. Secondly, the fixation systems were integrated with the cervical spine models without analyzing the interaction between the fixation systems and the bone-tissue interface post-implantations. Nonetheless, considering the design concepts and principles of different implants, they were placed in optimal positions to simulate their functional state, which was not expected to significantly impact the research outcomes. Thirdly, this study simulated all cervical spine models with various surgical treatment methods, overlooking the influence of surgical indications. However, this limitation was mitigated by standardized settings and statistical analysis of the cervical spine models, reducing the impact of individual patient differences on the research results. The research methodology employed in this study provides an intuitive representation of the biomechanical characteristics of different surgical treatment methods, making it visually reliable compared to traditional clinical studies. Therefore, despite certain factors that may affect its accuracy, the research methodology employed in this study effectively minimized the impact of confounding factors on the research conclusions, ensuring the reference value of the research results.

## 5 Conclusion

This study demonstrates that in the treatment of non-contiguous CDD, CDA offers significant biomechanical advantages and effectively avoids stress concentration in the middle and adjacent segments. On the other hand, skip-level ACDF significantly increase the stress burden on adjacent and middle segments, thereby elevating the risk of ASD, and is not recommended for non-contiguous CDD treatment. For cases where surgical indications permit, skip-level CDA is recommended as it achieves optimal biomechanical performance for the cervical spine. In situations where skip-level CDA is not feasible for a patient, Hybrid surgery can be considered. Among the Hybrid constructs, the FA construct demonstrates superior biomechanical performance compared to the AF construct. If both combinations are viable, the FA construct is recommended for treating non-contiguous CDD. Additionally, in cases where there is a notable difference in the degree of degeneration between segments, it is recommended to perform ACDF on the more severely degenerated segment, while CDA is suggested for the relatively less degenerated segment. Overall, the findings of this study provide valuable insights for clinicians when selecting the most appropriate surgical approach to achieve optimal biomechanical outcomes in the treatment of non-contiguous CDD.

## Data Availability

The raw data supporting the conclusions of this article will be made available by the authors, without undue reservation.

## References

[B1] Barbagallo G. M. Assietti R. Corbino L. Olindo G. Foti P. V. Russo V. (2009). Early results and review of the literature of a novel hybrid surgical technique combining cervical arthrodesis and disc arthroplasty for treating multilevel degenerative disc disease: opposite or complementary techniques? Eur. Spine J. 18 (1), 29–39. 10.1007/s00586-009-0978-9 19415346 PMC2899598

[B2] Barrey C. Campana S. Persohn S. Perrin G. Skalli W. (2012). Cervical disc prosthesis versus arthrodesis using one-level, hybrid and two-level constructs: an *in vitro* investigation. Eur. Spine J. 21, 432–442. 10.1007/s00586-011-1974-4 21833571 PMC3296854

[B3] Coric D. Nunley P. D. Guyer R. D. Musante D. Carmody C. N. Gordon C. R. (2011). Prospective, randomized, multicenter study of cervical arthroplasty: 269 patients from the kineflex|c artificial disc investigational device exemption study with a minimum 2-year follow-up: clinical article. J. Neurosurg. Spine 15, 348–358. 10.3171/2011.5.SPINE10769 21699471

[B4] Garay R. S. Solitro G. F. Lam K. C. Morris R. P. Albarghouthi A. Lindsey R. W. (2022). Characterization of regional variation of bone mineral density in the geriatric human cervical spine by quantitative computed tomography. Plos One 17, e0271187. 10.1371/journal.pone.0271187 35802639 PMC9269429

[B5] Goffin J. Van Calenbergh F. van Loon J. Casey A. Kehr P. Liebig K. (2003). Intermediate follow-up after treatment of degenerative disc disease with the bryan cervical disc prosthesis: single-level and bi-level. Spine (Phila Pa 1976) 28, 2673–2678. 10.1097/01.BRS.0000099392.90849.AA 14673368

[B6] Kaiser M. G. Haid R. J. Subach B. R. Barnes B. Rodts G. J. (2002). Anterior cervical plating enhances arthrodesis after discectomy and fusion with cortical allograft. Neurosurgery 50 (229-236), 229–238. 10.1097/00006123-200202000-00001 11844257

[B7] Kallemeyn N. Gandhi A. Kode S. Shivanna K. Smucker J. Grosland N. (2010). Validation of a c2-c7 cervical spine finite element model using specimen-specific flexibility data. Med. Eng. Phys. 32, 482–489. 10.1016/j.medengphy.2010.03.001 20392660

[B8] Laratta J. L. Shillingford J. N. Saifi C. Riew K. D. (2018). Cervical disc arthroplasty: a comprehensive review of single-level, multilevel, and hybrid procedures. Glob. Spine J. 8, 78–83. 10.1177/2192568217701095 PMC5810892 29456918

[B9] Lee J. H. Park W. M. Kim Y. H. Jahng T. A. (2016). A biomechanical analysis of an artificial disc with a shock-absorbing core property by using whole-cervical spine finite element analysis. Spine (Phila Pa 1976) 41, E893–E901. 10.1097/BRS.0000000000001468 26825785

[B10] Lee M. J. Dumonski M. Phillips F. M. Voronov L. I. Renner S. M. Carandang G. (2011). Disc replacement adjacent to cervical fusion: a biomechanical comparison of hybrid construct versus two-level fusion. Spine (Phila Pa 1976) 36, 1932–1939. 10.1097/BRS.0b013e3181fc1aff 21289581

[B11] Lee S. B. Cho K. S. Kim J. Y. Yoo D. S. Lee T. G. Huh P. W. (2012). Hybrid surgery of multilevel cervical degenerative disc disease: review of literature and clinical results. J. Korean Neurosurg. Soc. 52, 452–458. 10.3340/jkns.2012.52.5.452 23323165 PMC3539079

[B12] Li Y. Zhu J. Liao Z. Zhang Z. Liu W. (2018). Hybrid constructs for performing three-level hybrid surgery: a finite element study. World Neurosurg. 114, e1302–e1309. 10.1016/j.wneu.2018.03.202 29627629

[B13] Lin C. Y. Kang H. Rouleau J. P. Hollister S. J. Marca F. L. (2009). Stress analysis of the interface between cervical vertebrae end plates and the bryan, prestige lp, and prodisc-c cervical disc prostheses: an *in vivo* image-based finite element study. Spine (Phila Pa 1976) 34, 1554–1560. 10.1097/BRS.0b013e3181aa643b 19564765

[B14] Liu B. Zeng Z. Hoof T. V. Kalala J. P. Liu Z. Wu B. (2015). Comparison of hybrid constructs with 2-level artificial disc replacement and 2-level anterior cervical discectomy and fusion for surgical reconstruction of the cervical spine: a kinematic study in whole cadavers. Med. Sci. Monit. 21, 1031–1037. 10.12659/MSM.892712 25853772 PMC4403376

[B15] Liu Q. Guo Q. Yang J. Zhang P. Xu T. Cheng X. (2016). Subaxial cervical intradiscal pressure and segmental kinematics following atlantoaxial fixation in different angles. World Neurosurg. 87, 521–528. 10.1016/j.wneu.2015.09.025 26409072

[B16] Manickam P. S. Roy S. (2022). The biomechanical study of cervical spine: a finite element analysis. Int. J. Artif. Organs 45, 89–95. 10.1177/0391398821995495 33645324

[B17] Mo Z. Li Q. Jia Z. Yang J. Wong D. W. Fan Y. (2017). Biomechanical consideration of prosthesis selection in hybrid surgery for bi-level cervical disc degenerative diseases. Eur. Spine J. 26, 1181–1190. 10.1007/s00586-016-4777-9 27652678

[B18] Mo Z. Zhao Y. Du C. Sun Y. Zhang M. Fan Y. (2015). Does location of rotation center in artificial disc affect cervical biomechanics? Spine (Phila Pa 1976) 40, E469–E475. 10.1097/BRS.0000000000000818 25868102

[B19] Panjabi M. M. Crisco J. J. Vasavada A. Oda T. Cholewicki J. Nibu K. (2001). Mechanical properties of the human cervical spine as shown by three-dimensional load-displacement curves. Spine (Phila Pa 1976) 26, 2692–2700. 10.1097/00007632-200112150-00012 11740357

[B20] Qizhi S. Lei S. Peijia L. Hanping Z. Hongwei H. Junsheng C. (2016). A comparison of zero-profile devices and artificial cervical disks in patients with 2 noncontiguous levels of cervical spondylosis. Clin. Spine Surg. 29, E61–E66. 10.1097/BSD.0000000000000096 26889993

[B21] Shi S. Liu Z. D. You W. J. Ouyang Y. P. Li X. F. Qian L. (2016). Application of a stand-alone anchored spacer in noncontiguous anterior cervical arthrodesis with radiologic analysis of the intermediate segment. J. Clin. Neurosci. 25, 69–74. 10.1016/j.jocn.2015.05.050 26597607

[B22] Sun X. Sun S. Zhang T. Kong C. Wang W. Lu S. (2020). Biomechanical comparison of noncontiguous cervical disc arthroplasty and noncontiguous cervical discectomy and fusion in the treatment of noncontinuous cervical degenerative disc disease: a finite element analysis. J. Orthop. Surg. Res. 15, 36. 10.1186/s13018-020-1549-3 32005193 PMC6995191

[B23] Sun X. Zhang Q. Cao L. Wang J. Huang J. Liu Y. (2023). Biomechanical effects of hybrid constructions in the treatment of noncontinuous cervical spondylopathy: a finite element analysis. J. Orthop. Surg. Res. 18, 57. 10.1186/s13018-023-03537-7 36658557 PMC9854215

[B24] Tang B. Yang J. Zhang Y. Ren X. Jiang T. Mo Z. (2022). Incorporating strategy in hybrid surgery for continuous two-level cervical spondylosis from a biomechanical perspective. Comput. Methods Programs Biomed. 226, 107193. 10.1016/j.cmpb.2022.107193 36288687

[B25] Tohamy M. H. Osterhoff G. Abdelgawaad A. S. Ezzati A. Heyde C. E. (2022). Anterior cervical corpectomy and fusion with stand-alone cages in patients with multilevel degenerative cervical spine disease is safe. Bmc Musculoskelet. Disord. 23, 20. 10.1186/s12891-021-04883-5 34980062 PMC8725343

[B26] Wang K. F. Duan S. Zhu Z. Q. Liu H. Y. Liu C. J. Xu S. (2018). Clinical and radiologic features of 3 reconstructive procedures for the surgical management of patients with bilevel cervical degenerative disc disease at a minimum follow-up period of 5 years: a comparative study. World Neurosurg. 113, e70–e76. 10.1016/j.wneu.2018.01.157 29408574

[B27] Wu T. K. Meng Y. Liu H. Wang B. Y. Hong Y. Rong X. (2019a). Biomechanical effects on the intermediate segment of noncontiguous hybrid surgery with cervical disc arthroplasty and anterior cervical discectomy and fusion: a finite element analysis. Spine J. 19, 1254–1263. 10.1016/j.spinee.2019.02.004 30742975

[B28] Wu T. K. Meng Y. Wang B. Y. Rong X. Hong Y. Ding C. (2019b). Biomechanics following skip-level cervical disc arthroplasty versus skip-level cervical discectomy and fusion: a finite element-based study. Bmc Musculoskelet. Disord. 20, 49. 10.1186/s12891-019-2425-3 30704444 PMC6357490

[B29] Wu T. K. Wang B. Y. Cheng D. Rong X. Lou J. G. Hong Y. (2017a). Clinical and radiographic features of hybrid surgery for the treatment of skip-level cervical degenerative disc disease: a minimum 24-month follow-up. J. Clin. Neurosci. 40, 102–108. 10.1016/j.jocn.2017.02.030 28246009

[B30] Wu T. K. Wang B. Y. Deng M. D. Hong Y. Rong X. Chen H. (2017b). A comparison of anterior cervical discectomy and fusion combined with cervical disc arthroplasty and cervical disc arthroplasty for the treatment of skip-level cervical degenerative disc disease: a retrospective study. Med. Baltim. 96, e8112. 10.1097/MD.0000000000008112 PMC5662301 29019878

[B31] Xiong Y. Xu L. Yu X. Yang Y. Zhao D. Hu Z. (2018). Comparison of 6-year follow-up result of hybrid surgery and anterior cervical discectomy and fusion for the treatment of contiguous two-segment cervical degenerative disc diseases. Spine (Phila Pa 1976) 43, 1418–1425. 10.1097/BRS.0000000000002639 29547460

[B32] Yang Y. Ma L. Liu H. Liu Y. Hong Y. Wang B. (2016). Comparison of the incidence of patient-reported post-operative dysphagia between acdf with a traditional anterior plate and artificial cervical disc replacement. Clin. Neurol. Neurosurg. 148, 72–78. 10.1016/j.clineuro.2016.07.020 27428486

[B33] Zhao H. Cheng L. Hou Y. Liu Y. Liu B. Mundra J. J. (2015). Multi-level cervical disc arthroplasty (cda) versus single-level cda for the treatment of cervical disc diseases: a meta-analysis. Eur. Spine J. 24, 101–112. 10.1007/s00586-014-3429-1 24961223

